# Species-specific components of the *Helicobacter pylori* Cag type IV secretion system

**DOI:** 10.1128/iai.00493-24

**Published:** 2025-04-10

**Authors:** Kaeli N. Bryant, Arwen E. Frick-Cheng, Lauren E. Solecki, Heather K. Kroh, W. Hayes McDonald, D. Borden Lacy, Mark S. McClain, Melanie D. Ohi, Timothy L. Cover

**Affiliations:** 1 Department of Pathology, Microbiology, and Immunology, Vanderbilt University Medical Center 204907, Nashville, Tennessee, USA; 2 Life Sciences Institute, University of Michigan 123743, Ann Arbor, Michigan, USA; 3 Mass Spectrometry Research Center, Vanderbilt University School of Medicine 12327 , Nashville, Tennessee, USA; 4 Department of Biochemistry, Vanderbilt University 215875, Nashville, Tennessee, USA; 5 Veterans Affairs Tennessee Valley Healthcare System, Nashville, Tennessee, USA; 6 Vanderbilt Institute for Infection, Immunology, and Inflammation, Vanderbilt University Medical Center 12328, Nashville, Tennessee, USA; 7 Department of Medicine, Vanderbilt University School of Medicine 12327 , Nashville, Tennessee, USA; 8 Department of Cell and Developmental Biology, University of Michigan 1259, Ann Arbor, Michigan, USA; University of Pennsylvania Perelman School of Medicine, Philadelphia, Pennsylvania, USA

**Keywords:** *Helicobacter pylori*, bacterial protein secretion, bacterial secretion systems, proteomics

## Abstract

*Helicobacter pylori* strains containing the *cag* pathogenicity island (PAI) deliver an effector protein (CagA) and non-protein substrates into gastric cells through a process that requires the Cag type IV secretion system (T4SS). The Cag T4SS outer membrane core complex (OMCC) contains multiple copies of five proteins, two of which are species-specific proteins. By using modifications of a previously described OMCC immunopurification method and optimized mass spectrometric methods, we have now isolated additional *cag* PAI-encoded proteins that are present in lower relative abundance. Four of these proteins (CagW, CagL, CagI, and CagH) do not exhibit sequence relatedness to T4SS components in other bacterial species. Size exclusion chromatography analysis of immunopurified samples revealed that CagW, CagL, CagI, and CagH co-elute with OMCC components. These four Cag proteins are copurified with the OMCC in immunopurifications from a Δ*cag3* mutant strain (lacking peripheral OMCC components), but not from a Δ*cagX* mutant strain (defective in OMCC assembly). Negative stain electron microscopy analysis indicated that OMCC preparations isolated from Δ*cagW, cagL::kan,* Δ*cagI, and* Δ*cagH* mutant strains are indistinguishable from wild-type OMCCs. In summary, by using several complementary methods, we have identified multiple species-specific Cag proteins that are associated with the Cag T4SS OMCC and are required for T4SS activity.

## INTRODUCTION

*Helicobacter pylori* is a gram-negative, spiral-shaped bacterium that colonizes the gastric mucosa of roughly half of the world population (1). Most colonized individuals remain asymptomatic, but a small percentage develop peptic ulcers or gastric cancer (2–7). *H. pylori* is the primary risk factor for the development of gastric cancer and has been classified as a Group I carcinogen by the World Health Organization (8). The risk for the development of gastric cancer or peptic ulcers in *H. pylori*-colonized individuals is dependent on several factors, including host genetic characteristics, diet, environmental exposures, and *H. pylori* strain-specific features (9–13).

One of the *H. pylori* strain-specific features that influences the risk of gastric cancer is a 40 kb genomic region known as the *cag* pathogenicity island (PAI) (13–15). *H. pylori* strains that possess the *cag* PAI are associated with significantly higher rates of gastric cancer than strains that lack this region (10, 16–18). The *cag* PAI encodes the Cag type IV secretion system (T4SS) (15, 19–22) as well as CagA, a secreted effector protein (23, 24). CagA is translocated into gastric epithelial cells via the Cag T4SS, causing cellular alterations linked to carcinogenesis (23, 24). Therefore, CagA has been designated as a bacterial oncoprotein. Although CagA is the only known protein secreted by the Cag T4SS, several types of non-protein substrates are delivered into host cells through Cag T4SS-dependent processes. These include LPS metabolites (e.g. ADP-heptose), peptidoglycan, and DNA. These non-protein secreted substrates are recognized by sensors within host cells, leading to the activation or repression of cellular signaling (25–31).

Prototypical T4SSs contain 12 different proteins, designated as VirB1-11 and VirD4 (32, 33). Most of these proteins are localized within two major T4SS subassemblies: the inner membrane complex (IMC) and the outer membrane core complex (OMCC) (34–38). Additional structural features, including the stalk and arches, span the periplasm to connect the OMCC and IMC (39, 40). These structural features of bacterial T4SSs have been defined using single-particle cryo-electron microscopy (cryo-EM) or cryo-electron tomography (cryo-ET) (39, 41). In the *E. coli* R388 conjugation system (a prototypical T4SS), VirB7, VirB9, and VirB10 are localized to the OMCC; VirB5 and VirB6 are localized to the stalk; VirB8 is localized to the arches; and VirB3, VirB4, VirB11, and VirD4 are localized to the IMC (33, 39, 40, 42).

The *H. pylori* Cag T4SS is classified as an “expanded” T4SS, as at least 17 proteins encoded by the *cag* PAI are required for Cag T4SS activity (15, 22, 32, 37, 43). Single particle cryo-EM analysis of the Cag T4SS OMCC revealed that it contains five protein components: Cag3, CagY, CagX, CagT, and CagM (34, 36). The C-terminal portions of CagY and CagX are structurally similar to VirB10 and VirB9 homologs, respectively (34). CagT is a lipoprotein that exhibits structural relatedness to VirB7 homologs within the N-terminal region (20, 21, 34, 44, 45). Cag3 and CagM do not share sequence or structural relatedness to T4SS components in other bacterial species (34, 36). Comparative analyses of the Cag T4SS IMC in wild-type and mutant *H. pylori* strains by cryo-ET indicated that the IMC contains at least three protein components: Cagα, Cagβ, and CagE (VirB11, VirD4, and VirB3/4, respectively) (35, 37).

Each of the known components of the *H. pylori* Cag T4SS OMCC and IMC is required for the translocation of CagA by the Cag T4SS (43, 46, 47). At least eight additional *cag* PAI-encoded proteins (hereafter termed “Cag proteins”) are also required for activity; some are related to VirB components of T4SSs from other bacterial species, whereas others are unrelated to VirB proteins and are only found in *H. pylori* (15, 20, 22, 43, 48). Furthermore, there are several *H. pylori* proteins not encoded by *cag* PAI (e.g., HopQ, BabA, AlpA/B, Hyd, and HP1564) that are reportedly required for Cag T4SS activity (49–56). The mechanisms by which these latter proteins (hereafter termed “non-Cag proteins”) contribute to Cag T4SS activity are not known.

In this study, we report the use of a previously described OMCC immunopurification method (targeting epitope-tagged CagF) and optimized mass spectrometric methods to isolate the five known OMCC components of the *H. pylori* Cag T4SS along with additional Cag proteins that are associated with the OMCC (34, 36, 46). These results provide new insights into species-specific components of the Cag T4SS that are required for Cag T4SS activity.

## RESULTS

### Isolation of Cag proteins using HA-CagF as a bait

Previous studies demonstrated that hemagglutinin-tagged CagF (HA-CagF) can be used as a bait for isolating CagA and five protein components of the *H. pylori* Cag T4SS outer membrane core complex (OMCC) (34, 36, 46). In an effort to isolate additional components of the Cag T4SS, we modified multiple steps in the immunopurification protocol, including culturing larger volumes of *H. pylori*, adjusting the bacterial lysis conditions and optimizing the immunopurification conditions, and then using more sensitive mass spectrometry detection methods as described in the Methods section. In experiments conducted using these modifications, we noted that multiple additional Cag proteins (Cag1, Cagβ, Cagα, CagZ, CagW, CagV, CagS, CagN, CagL, CagI, CagH, CagG, CagE, and CagD) were consistently detected in the immunopurified preparations, along with the previously identified HA-CagF, CagA, and five OMCC protein components (Table S1).

To evaluate if the isolation of these additional Cag proteins was specifically dependent on the use of HA-CagF as a target for the immunopurification, we analyzed two control strains engineered to produce HA-tagged proteins that are not considered part of the Cag T4SS: HP0179-HA (produced by strain “VM329”), a predicted LolD protein that has a role in lipoprotein trafficking; and HP0838-HA (produced by strain “VM333”), a predicted lipoprotein of unknown function (57). Previous studies confirmed that these strains produce the epitope-tagged proteins (HP0179-HA or HP0838-HA) and these proteins do not interact with the Cag T4SS (57). The strains producing HP0179-HA and HP0838-HA were grown, lysed, and analyzed following the same protocol used for isolation of the Cag T4SS OMCC with HA-CagF.

Mass spectrometry analysis showed that the five known OMCC proteins (Cag3, CagY, CagX, CagT, and CagM), CagA, and CagF were isolated from the HA-CagF strain, as expected (46). In contrast, these proteins were either not isolated or isolated at very low levels from the HP0179-HA and HP0838-HA control strains. Specifically, spectral counts of the five OMCC proteins, CagA, and CagF were undetected or were about 10-fold to 20-fold lower in preparations from the HP0179-HA and HP0838-HA control strains than from the HA-CagF strain (Table S1). Nine additional Cag proteins were isolated at higher levels from the HA-CagF strain than from the control strains: Cagβ, CagW, CagN, CagL, CagI, CagH, CagG, CagE, and CagD (Table S1). To assess the specificity and statistical significance of these results, we performed an unbiased SAINTexpress analysis on data from multiple immunopurifications of HA-CagF, HP0179-HA, and HP0838-HA (58, 59). By comparing the abundance of prey proteins from affinity purifications of test (i.e., HA-CagF) and control (i.e., HP0179-HA and HP0838-HA) bait proteins under identical conditions, SAINTexpress computes the probability that a prey protein identified in an immunopurified sample is a true interaction partner of the bait protein (i.e., a true interactor) or if the prey protein is a non-specific binder or contaminant (i.e., false interactor) (58, 59).

**Fig 1:**
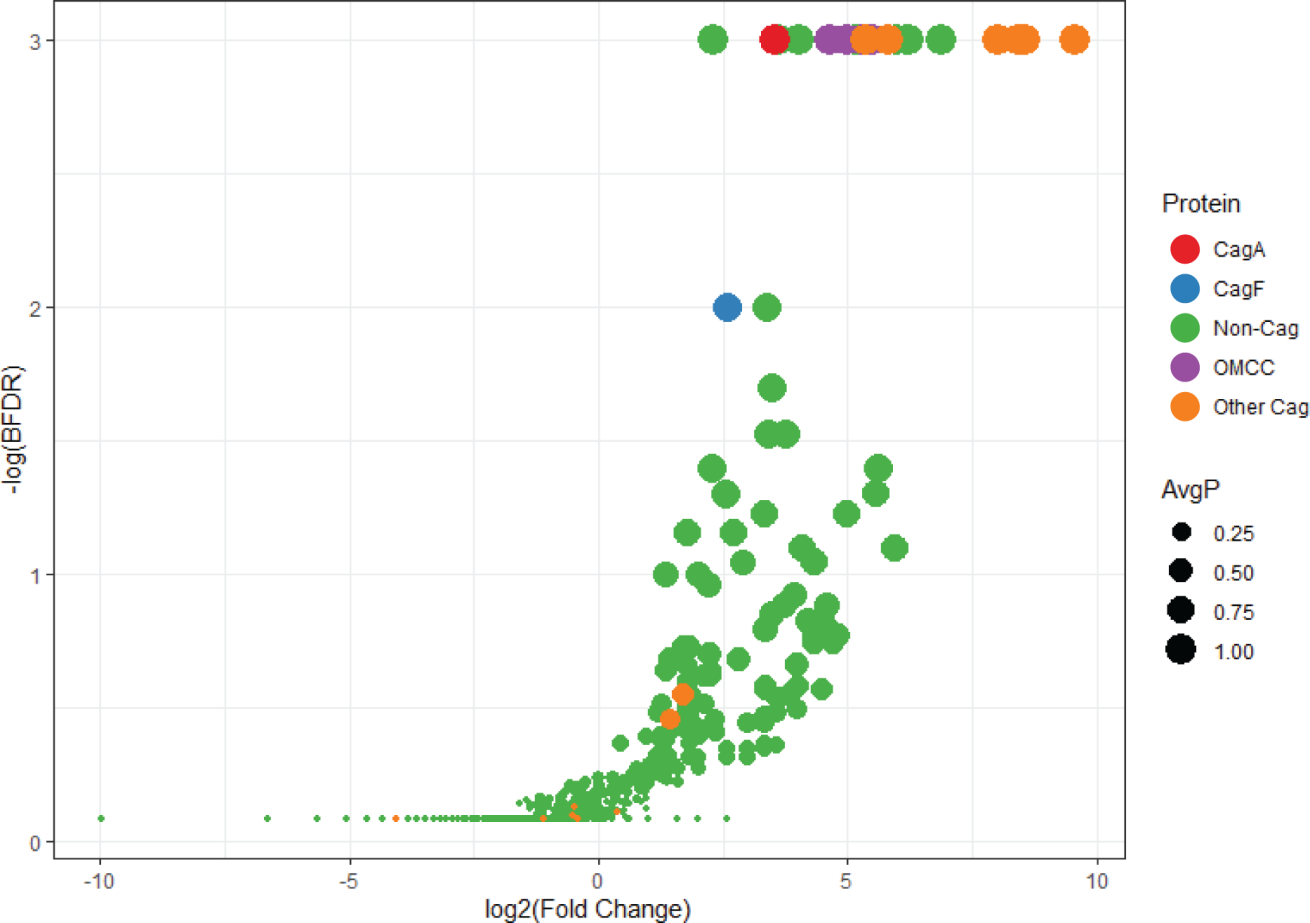
Volcano plot of HA-CagF interactions compared with protein interactions of HA-tagged control proteins. Immunopurified preparations from an HA-CagF-producing strain and from strains producing HA-tagged non-Cag control proteins (HP0179-HA, HP0838-HA) were analyzed by mass spectrometry, and the results were compared using SAINTexpress. Differences in the detected protein-protein interactions were assessed based on analysis of the Bayesian false discovery rate (−log(BFDR)) and fold change (log_2_(Fold Change)), with average interaction probability (AvgP) represented by dot size. A value of 0.001 was added to BFDR values calculated as 0 by SAINTexpress to allow for graphical analysis. Specific groups of interacting proteins are identified based on the color code. Twelve Cag proteins (CagA, the five OMCC components, and six newly isolated Cag proteins) had BFDR scores of 0. Detailed data pertaining to individual Cag proteins are shown in Table 1, and the complete data for all immunopurifications are shown in Table S1.

The results confirmed that the isolation of CagW, CagN, CagL, CagI, CagH, and CagG is significantly associated with the HA-CagF immunopurification (Table 1; Fig. 1). Several non-Cag proteins (encoded by genes located outside the *cag* PAI) were also isolated at significantly higher levels from the HA-CagF strain than from the control strains (Fig. 1; Table S1). These are considered in further detail in subsequent sections and in the Discussion.

**TABLE 1:** SAINTexpress analysis of Cag proteins associated with the HA-CagF immunopurification

Identified proteins^*a*^	Protein	AvgP^*b*^	log_2_(Fold Change)	−log(BFDR)^*c*^
CagA	CagA	1	3.53	3
Cag3	OMCC	1	5.51	3
CagY	OMCC	1	5.29	3
CagX	OMCC	1	4.98	3
CagM	OMCC	1	4.97	3
CagT	OMCC	1	4.64	3
CagI	Other Cag	1	9.57	3
CagH	Other Cag	1	8.57	3
CagL	Other Cag	1	8.43	3
CagN	Other Cag	1	8	3
CagG	Other Cag	1	5.81	3
CagW	Other Cag	1	5.35	3
CagF	CagF	0.84	2.58	2
CagD	Other Cag	0.39	1.68	0.55
CagE	Other Cag	0.35	1.43	0.46
Cagβ	Other Cag	0	0.39	0.11
CagV	Other Cag	0	−0.42	0.08
Cag1	Other Cag	0	−0.49	0.12
Cagα	Other Cag	0	−0.51	0.10
CagS	Other Cag	0	−1.12	0.08
CagZ	Other Cag	0	−4.06	0.08

^*a*^ HA-CagF immunopurification samples were compared to immunopurifications of non-Cag proteins (HP0179-HA, HP0838-HA) using SAINTexpress. All detected Cag proteins (encoded by genes in the *cag* PAI) are shown in this table. See Fig. 1 for graphical presentation. For data on all *H. pylori* proteins, see Table S1.

^*b*^ AvgP = probability of interaction with bait protein (HA-CagF). Values of 1 indicate a high probability of interaction.

^*c*^ BFDR = Bayesian false discovery rate.

To further investigate the specificity of the HA-CagF immunopurification, we modified a second wild-type strain of *H. pylori*, G27, to express HA-CagF. Mass spectrometry analysis showed that we isolated similar amounts of OMCC protein components from the engineered G27 strain, compared with the previously used HA-CagF-producing strain 26695 (Table 2). For additional comparisons, we generated *H. pylori* strains in the 26695 background that produced alternate HA-tagged Cag proteins: HA-Cagβ, HA-Cagα, CagZ-HA, CagY-HA, HA-CagE, and HA-CagA. Immunopurification experiments with these strains either did not result in isolation of OMCC components or were relatively ineffective in yielding OMCC components, in contrast to the immunopurifications targeting HA-CagF (Table S2; Table 2). However, these experiments corroborated several previously reported protein-protein interactions, including interactions between Cagβ and CagZ (detected here when targeting either Cagβ or CagZ) (60, 61) and interactions between CagF and CagA (detected here when targeting either CagF or CagA) (Table S2; Table 2) (62–64). Notably, the copurification of OMCC protein components was relatively inefficient when targeting HA-CagA, despite CagA being required for isolation of the OMCC when targeting HA-CagF (46). We confirmed that HA-CagA was translocation-competent by performing a CagA translocation assay, as described in the Methods section (Fig. S1).

**TABLE 2:** LC-MS/MS analysis of Cag proteins immunopurified from HA-CagF 26695 or HA-CagF G27

Identified proteins^*a*^	HA-CagF 26695^*b*^	HA-CagF G27^*b*^
Cag3^*c*^	1,378	583
CagY^*c*^	1,964	620
CagX^*c*^	1,172	512
CagT^*c*^	583	347
CagM^*c*^	874	405
CagF	1,000	928
CagA	4,027	2,133
CagW^*d*^	107	71
CagL^*d*^	34	15
CagI^*d*^	67	53
CagH^*d*^	68	36
Cag1	1	0
Cagβ	15	2
Cagα	4	9
CagV	25	23
CagG	2	2
CagE	27	19
CagD	8	12
No. of Cag spectra	11,366 (51%)	5,774 (43%)
No. of non-Cag spectra	10,836 (49%)	7,706 (57%)
Total spectral counts	22,202	13,480

^*a*^ Cag proteins were detected by mass spectrometry.

^*b*^ HA-CagF was isolated via immunopurification from the indicated strains as described in the Methods section. Numbers of spectral counts for the indicated proteins are shown.

^*c*^ Known outer membrane core complex (OMCC) proteins.

^*d*^ Newly isolated Cag proteins.

### Enrichment of Cag proteins in the HA-CagF immunopurification

**Fig 2:**
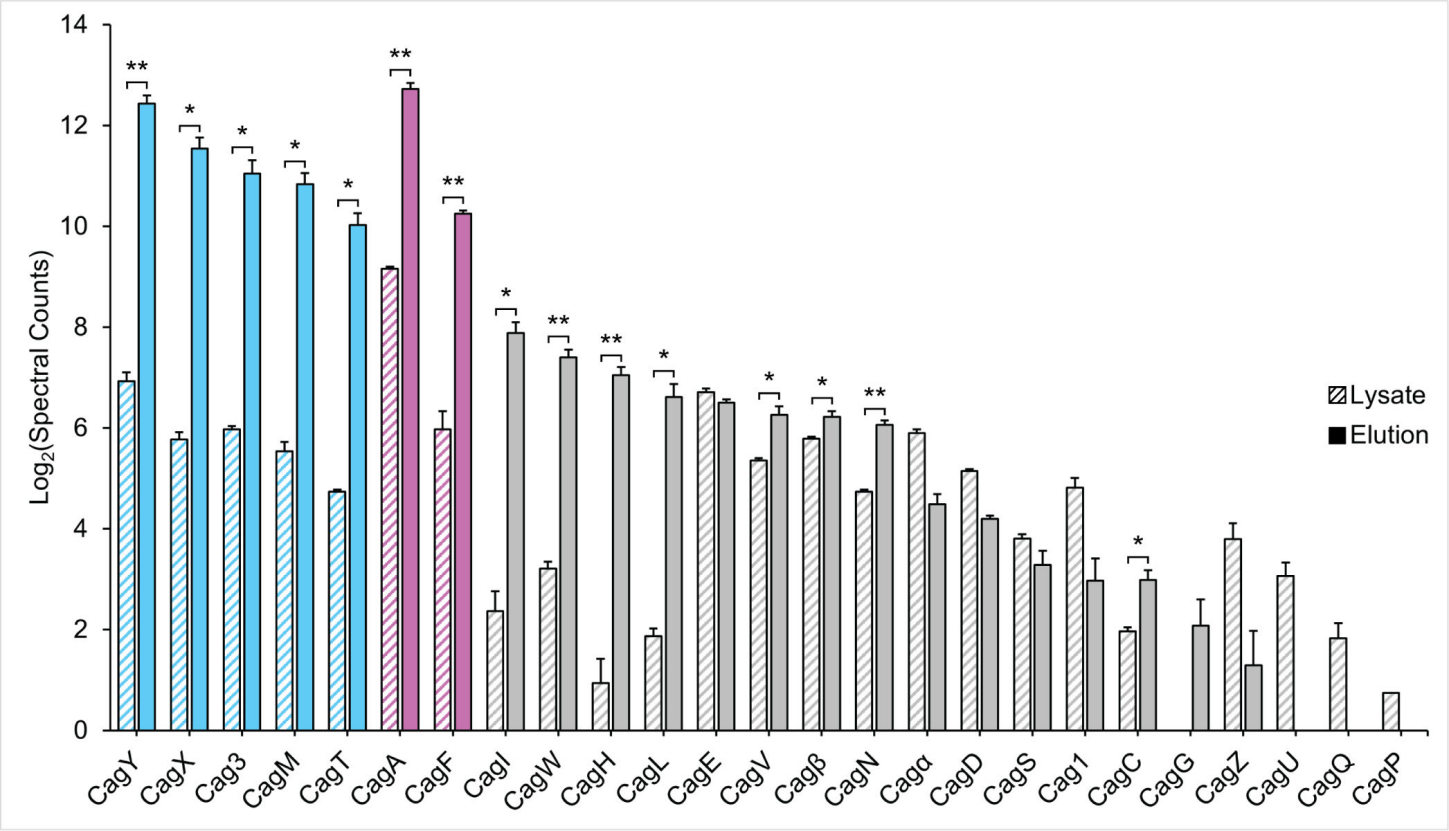
Cag proteins enriched in the HA-CagF immunopurification elution compared with unprocessed *H. pylori* lysate. The protein contents of *H. pylori* lysates and elution samples from HA-CagF immunopurifications (three biologically independent replicates) were analyzed by mass spectrometry. For each replicate, the spectral counts of the lysate and the immunopurification elution sample were normalized; data were log_2_-transformed, and averages for each protein were plotted. A value of 1 was added to the spectral count for each protein prior to log_2_ transformation to allow for graphical analysis of samples where no spectral counts were detected. Statistical significance was determined by comparing lysate and elution spectral counts for each Cag protein (paired *t*-test). *, *P* < 0.05; **, *P* < 0.01. OMCC components, blue; CagA and CagF, pink; other Cag proteins, gray.

As another experimental approach for identifying Cag proteins that were specifically isolated by targeting HA-CagF, we performed paired mass spectrometry analysis on unprocessed lysates from the HA-CagF-producing strain and samples immunopurified from these lysates (Table S3). This approach allowed us to identify Cag proteins that are enriched in the HA-CagF immunopurification elution compared with the unprocessed *H. pylori* lysate (i.e., increased relative abundance in the immunopurified sample compared with the starting material). As expected, this analysis showed that the immunopurification yielded markedly enriched levels of CagF, CagA, and the five OMCC components (Fig. 2). This analysis also identified eight additional Cag proteins that were significantly enriched in the immunopurified samples (Fig. 2), including several Cag proteins identified in the previous analysis (CagW, CagN, CagL, CagI, and CagH) (Fig. 1; Table 1). Other Cag proteins were not significantly enriched (e.g., CagE, CagS), were not detected in the elution (e.g., CagQ, CagP), or were not detected in either the lysate or elution (Cag2, Cag4) (Table S3). Several non-Cag proteins were enriched in the HA-CagF immunopurification, but only eight of these (HP0030, HP0282, HP0437, HP0438, HP0583, HP0747, HP1050, and HP1279) were also specific to the HA-CagF immunopurification (see Discussion for further details) (Table S1 and S3).

### CagW, CagL, CagI, and CagH associate with the OMCC

**Fig 3:**
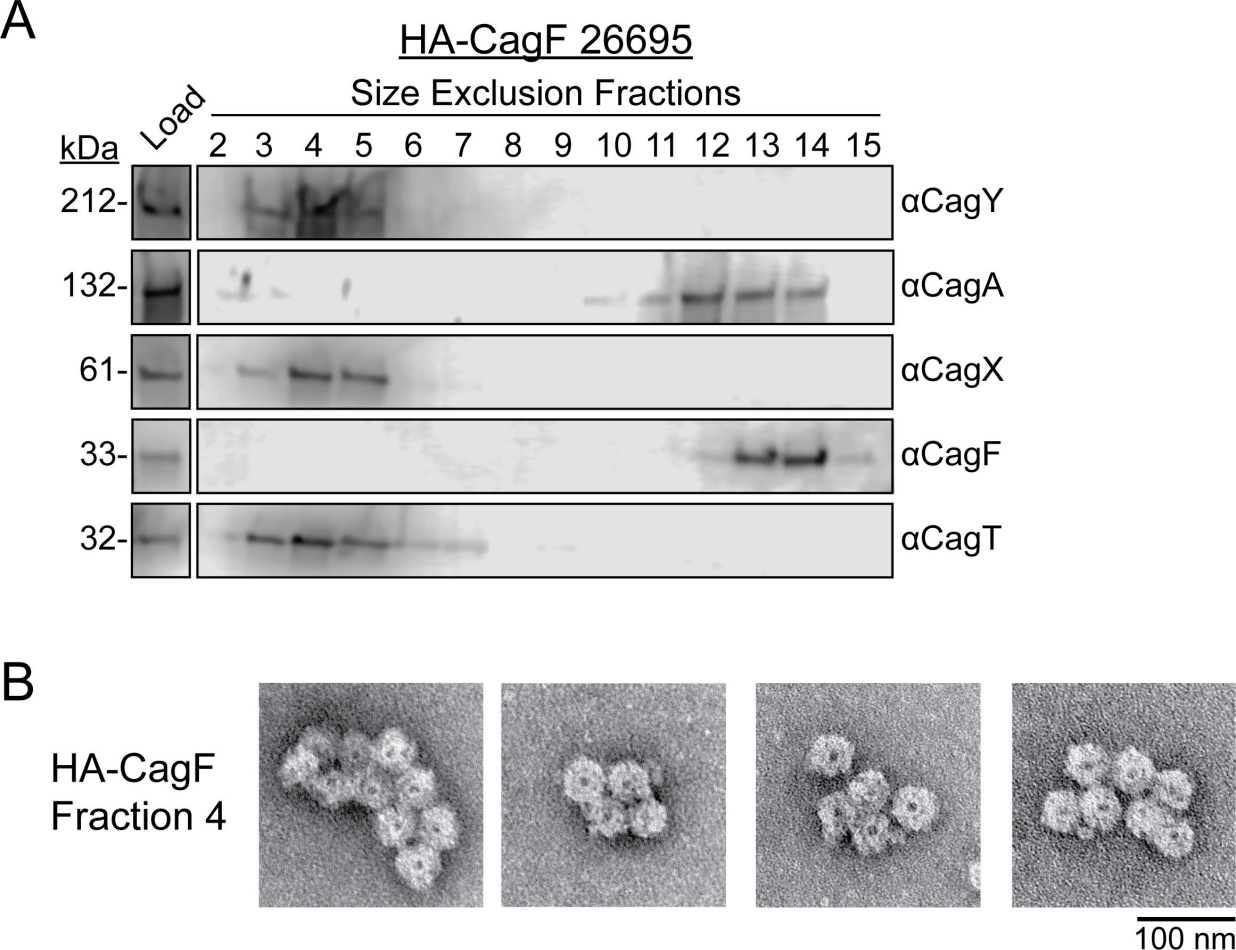
Size exclusion chromatography of the HA-CagF immunopurified sample. An HA-CagF immunopurification was performed, and the elution sample (labeled “Load”) was fractionated by size exclusion chromatography. (**A**) Fractions were analyzed by Western blotting using anti-CagY, CagA, CagX, CagF, or CagT antisera. (**B**) Cag T4SS outer membrane core complexes from fraction 4 were visualized by negative stain electron microscopy. Scale bar, 100 nm.

After finding that multiple Cag proteins are specifically isolated and enriched by the HA-CagF immunopurification, we then investigated if these proteins are physically associated with the OMCC. To do this, we used size exclusion chromatography to fractionate proteins isolated by the HA-CagF immunopurification. As expected, based on the large size of the OMCC, OMCC proteins were detected in the highest molecular mass fractions (void volume), and OMCC particles were visualized in these fractions by negative stain electron microscopy (NS-EM) (Fig. 3). In contrast, CagA and CagF eluted in lower molecular mass fractions, a result consistent with previous experiments using glycerol gradient analysis (Fig. 3A) (46). Due to low protein abundance, the absorbance at 280 nm was below the limit of detection for the FPLC, and no peaks were observed in the chromatogram.

**Fig 4:**
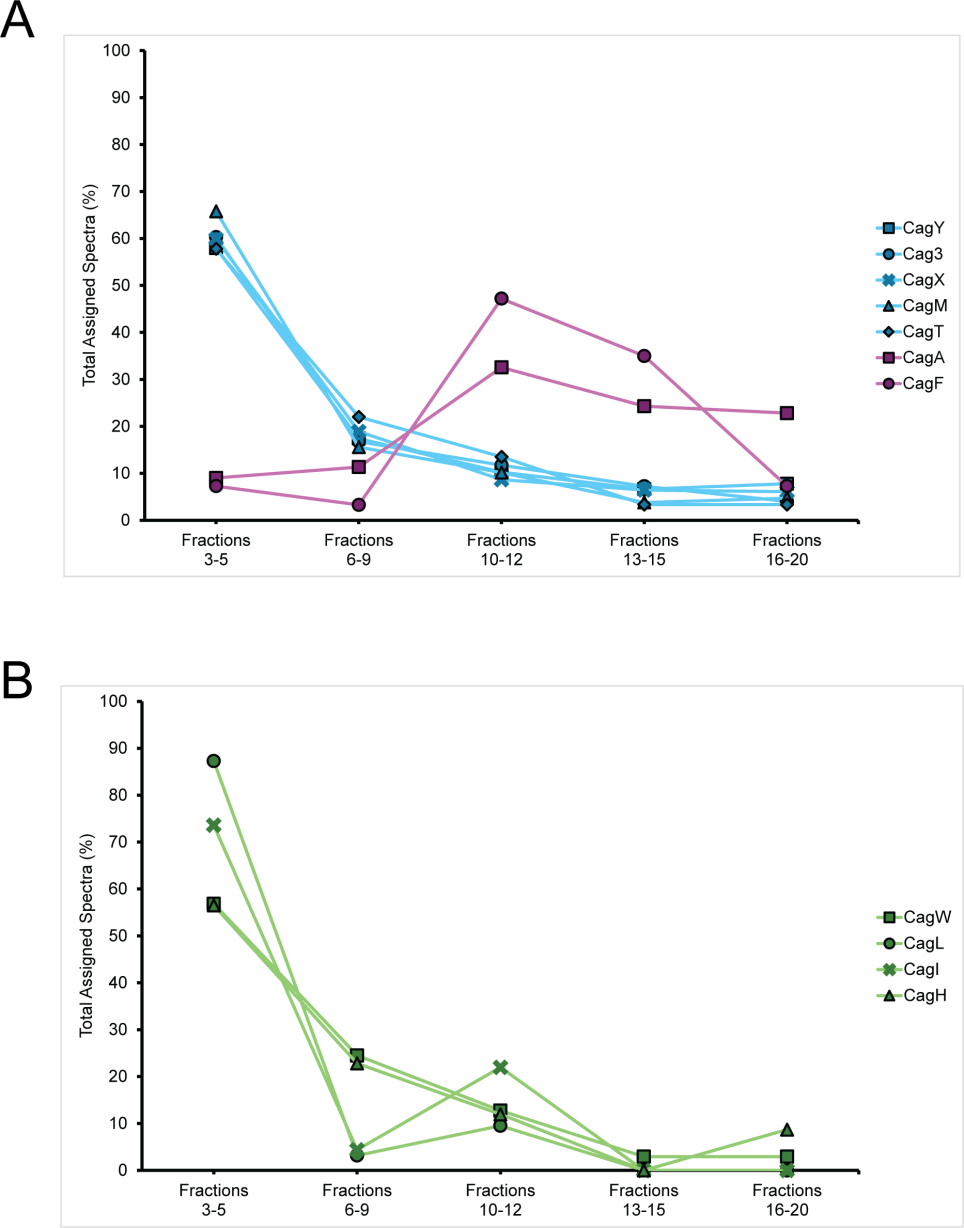
Mass spectrometry analysis of size exclusion chromatography fractions. An HA-CagF immunopurification was performed, and the elution sample was fractionated by size exclusion chromatography. Fractions were pooled as indicated for mass spectrometry analysis. (**A**) Percentages of total spectral counts for the five outer membrane core complex proteins (blue) or CagA and CagF (pink) in pooled size exclusion chromatography fractions are shown. (**B**) Percentages of total spectral counts for newly isolated Cag proteins (CagW, CagL, CagI, and CagH) in pooled size exclusion chromatography fractions are shown.

As a next step, pooled fractions were analyzed by mass spectrometry, and we assessed the distribution of spectral counts for each protein of interest among the pooled fractions. This analysis confirmed that the five OMCC protein components are most abundant in fractions corresponding to the void volume, whereas CagA and CagF are most abundant in lower molecular mass fractions (Table S4; Fig. 4A). CagW, CagL, CagI, and CagH were predominantly detected in the same high molecular mass fractions as the OMCC proteins (Table S4; Fig. 4B). Analysis of CagN and CagG was limited by the detection of very low numbers of spectral counts for these two proteins (Table S4). Several non-Cag proteins were detected in the void volume, but most were not found to be associated with HA-CagF in the previous immunopurification experiments and are therefore not considered specifically associated with the OMCC. Only one of these non-Cag proteins (HP0282, an RCK C-terminal domain-containing protein) was specifically associated with HA-CagF in the previous immunopurification experiments (see Discussion for further details) (Tables S1 and S3).

### Isolation of CagW, CagL, CagI, and CagH is dependent on the presence of the OMCC

To determine if HA-CagF-dependent isolation of CagW, CagL, CagI, or CagH is dependent on the presence of the OMCC, we analyzed an *H. pylori* strain that fails to assemble an OMCC (HA-CagF Δ*cagX*) and an *H. pylori* strain from which OMCC components cannot be isolated using HA-CagF (HA-CagF Δ*cagA*) (46). Consistent with expectations, the HA-CagF Δ*cagX* immunopurification sample contained CagF and CagA, and the HA-CagF Δ*cagA* immunopurification sample contained CagF (Table 3) (46). The HA-CagF Δ*cagX* and HA-CagF Δ*cagA* immunopurified samples contained minimal amounts of the five known OMCC proteins, corresponding to non-specific isolation (Table 3). Additionally, we were unable to visualize purified OMCCs from these strains by NS-EM (46). CagW, CagL, CagI, and CagH were not isolated from the HA-CagF Δ*cagX* strain or from the HA-CagF Δ*cagA* strain (Table 3). We confirmed that CagW, CagL, CagI, and CagH were stably produced by the parental wild-type strain (HA-CagF) and the mutant strains by analyzing unprocessed lysates using mass spectrometry (Table S5). These experiments provide further evidence that the isolation of CagW, CagL, CagI, and CagH is dependent on the presence of the Cag T4SS OMCC (Table 3).

**TABLE 3:** LC-MS/MS analysis of proteins immunopurified from the indicated strains

Identified proteins^*a*^	WT/HA-CagF^*b*^	Δcag3/HA-CagF^*b*^	ΔcagM/HA-CagF^*b*^	ΔcagX/HA-CagF^*b*^	ΔcagA/HA-CagF^*b*^
Cag3^*c*^	2,670	0	14	2	0
CagY^*c*^	3,147	4,358	973	32	3
CagX^*c*^	1,365	1,706	452	0	0
CagT^*c*^	1,189	1,353	11	0	0
CagM^*c*^	1,615	1,833	1	0	1
CagF	856	1,591	512	1,047	791
CagA	6,574	9,700	3,524	7,803	0
CagW^*d*^	61	15	42	**0**	**1**
CagL^*d*^	15	7	31	**0**	**0**
CagI^*d*^	19	17	49	**0**	**0**
CagH^*d*^	19	4	47	**0**	**0**
Cag1	2	1	2	3	2
Cagβ	26	11	2	1	0
Cagα	5	0	0	12	0
CagZ	0	1	0	0	3
CagV	7	15	23	14	19
CagN	5	4	3	0	0
CagG	3	0	0	0	0
CagE	9	9	10	15	7
CagD	5	1	12	15	0
No. of Cag spectra	17,592 (77%)	20,626 (88%)	5,710 (42%)	8,945 (62%)	827 (10%)
No. of non-Cag spectra	5,217 (23%)	2,943 (12%)	7,763 (58%)	5,472 (38%)	7,255 (90%)
Total spectral counts	22,809	23,569	13,473	14,417	8,082

^*a*^ Cag proteins were detected by mass spectrometry.

^*b*^ HA-CagF was isolated via immunopurification from the indicated strains as described in the Methods section. Numbers of spectral counts for the indicated proteins are shown. Results of interest are bolded and underlined.

^*c*^ Known outer membrane core complex (OMCC) proteins.

^*d*^ Newly isolated Cag proteins.

We performed a similar experiment with an HA-CagF Δ*cag3* mutant strain, which produces an OMCC lacking peripheral Cag3 components, and an HA-CagF Δ*cagM* mutant strain, which is only able to assemble the periplasmic ring (36, 38, 46). As expected, the immunopurified sample from the Δ*cag3* mutant contained four OMCC proteins (excluding Cag3), and the Δ*cagM* mutant immunopurified sample contained primarily CagY and CagX (Table 3) (36, 38, 46). We were able to detect CagW, CagL, CagI, and CagH in immunopurified samples from both the Δ*cag3* and Δ*cagM* mutants, suggesting that assembly of the outer membrane cap is not required for these proteins to interact with the OMCC (Table 3).

### CagW, CagL, CagI, and CagH are not required for OMCC assembly

**Fig 5:**
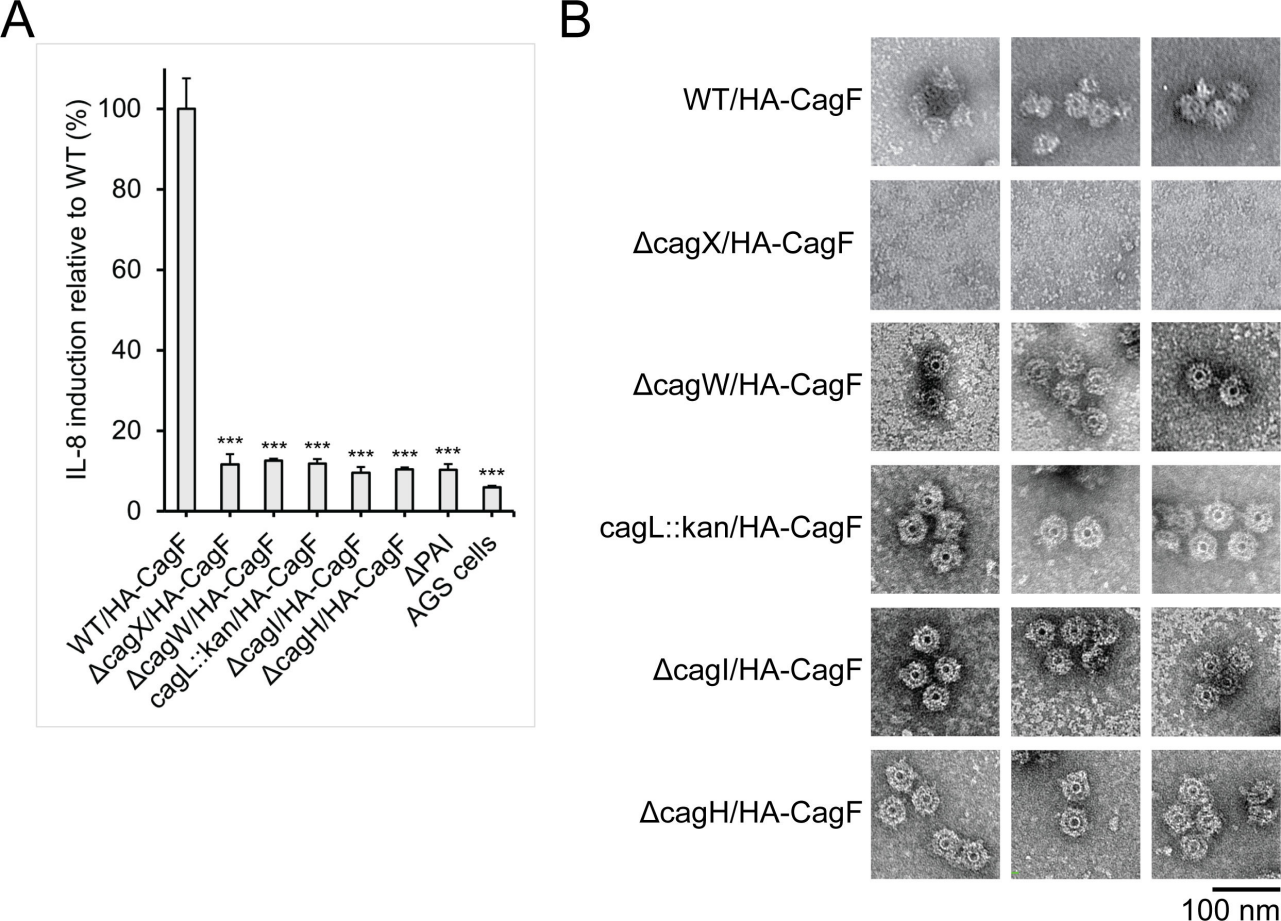
Functional properties of CagW, CagL, CagI, and CagH. (**A**) The indicated strains were co-cultured with AGS cells, and an IL-8 induction assay was performed as described in the Methods section. Statistically significant differences in the IL-8-inducing activities of mutant strains compared with the WT/HA-CagF strains were determined by a one-way ANOVA with Dunnett’s multiple comparison test. Results are from two biologically independent experiments. ***, *P* < 0.001. (**B**) Cag T4SS outer membrane core complexes were isolated from the indicated strains via immunopurification as described in the Methods section. Complexes were visualized by negative stain electron microscopy. Scale bar, 100 nm.

To define the functional properties of CagW, CagL, CagI, and CagH, we used previously described mutant strains in which *cagL, cagI*, or *cagH* were deleted (65) and also generated a Δ*cagW* mutant. Then, we modified each of the strains to allow the production of HA-CagF (HA-CagF Δ*cagW*, HA-CagF *cagL*::kan, HA-CagF Δ*cagI*, or HA-CagF Δ*cagH*). To evaluate the contributions of CagW, CagL, CagI, and CagH to the activity of the Cag T4SS, we analyzed the capacity of each of the strains to stimulate the production of IL-8 by AGS gastric epithelial cells. Cag T4SS-mediated stimulation of IL-8 production is dependent on the entry of LPS metabolites (such as ADP-heptose) into gastric epithelial cells (27–30). Consistent with previously reported results (43, 65), we found that these mutant strains were unable to induce IL-8 production in AGS cells (Fig. 5A).

Next, to evaluate if these proteins were required for OMCC assembly, we performed immunopurifications on the Δ*cagW*, *cagL::kan*, Δ*cagI*, or Δ*cagH* mutant strains, each modified to produce HA-CagF. NS-EM analysis of immunopurified preparations from the HA-CagF Δ*cagW*, HA-CagF *cagL*::kan, HA-CagF Δ*cagI*, and HA-CagF Δ*cagH* mutant strains showed particles that were indistinguishable from the wild-type OMCC (Fig. 5B) (46). We were unable to isolate particles from the HA-CagF Δ*cagX* mutant strain, which has been previously shown to be defective in OMCC assembly (Fig. 5B) (35, 46). These results indicate that OMCC assembly does not require CagW, CagL, CagI, or CagH, in contrast to proteins such as CagY or CagX that are required for OMCC assembly (35, 46).

We also performed mass spectrometry analysis on samples immunopurified from the HA-CagF Δ*cagW*, HA-CagF *cagL*::kan, HA-CagF Δ*cagI*, or HA-CagF Δ*cagH* strains. The five OMCC protein components were successfully isolated from each mutant strain, as expected based on NS-EM analysis (Table 4; Fig. 5B). Similarly, CagI, CagH, and CagL or CagW were successfully isolated from the Δ*cagW* or *cagL::kan* mutant strains, respectively (Table 4). Conversely, CagL was not isolated from the Δ*cagH* mutant, and CagW, CagL, and CagH were not isolated from the Δ*cagI* mutant (Table 4). We confirmed that CagW, CagL, CagI, and CagH were present in the unprocessed lysates of the HA-CagF Δ*cagI* and HA-CagF Δ*cagH* mutant strains (except when the relevant gene was deleted), using mass spectrometry (Table S5). Collectively, these results provide evidence that CagW, CagL, CagI, and CagH are associated with the Cag T4SS OMCC but are not required for OMCC assembly.

**TABLE 4:** LC-MS/MS analysis of proteins immunopurified from the indicated strains

Identified proteins^*a*^	ΔcagW/HA-CagF^*b*^	cagL::kan/HA-CagF^*b*^	ΔcagI/HA-CagF^*b*^	ΔcagH/HA-CagF^*b*^
CagW^*c*^	**0**	17	**0**	14
CagL^*c*^	17	**0**	**0**	**0**
CagI^*c*^	30	11	**0**	9
CagH^*c*^	24	11	**0**	**0**
Cag3^*d*^	923	2,044	1,783	2,061
CagY^*d*^	1,261	2,337	1,836	2,317
CagX^*d*^	737	1,202	1,058	1,230
CagT^*d*^	303	816	570	826
CagM^*d*^	465	916	761	941
CagF	973	844	830	729
CagA	4,175	4,017	4,564	3,846
Cag1	1	2	3	4
Cagβ	13	27	22	30
Cagα	12	3	23	6
CagV	27	33	25	35
CagS	2	2	4	2
CagN	2	22	13	21
CagG	4	11	2	20
CagE	20	48	39	58
CagD	15	12	29	3
No. of Cag spectra	9,004 (43%)	12,375 (43%)	11,562 (45%)	12,152 (41%)
No. of non-Cag spectra	11,933 (57%)	16,296 (57%)	14,173 (55%)	17,101 (59%)
Total spectral counts	20,937	28,671	25,735	29,253

^*a*^ Cag proteins were detected by mass spectrometry.

^*b*^ HA-CagF was isolated via immunopurification from the indicated strains as described in the Methods section. Numbers of spectral counts for the indicated proteins are shown. Results of interest are bolded and underlined.

^*c*^ Newly isolated Cag proteins.

^*d*^ Known outer membrane core complex (OMCC) proteins.

## DISCUSSION

The *cag* PAI contains at least 17 genes that are required for Cag T4SS activity (15, 20, 22, 43). Some of these genes encode homologs of VirB/VirD4 proteins, but others encode proteins found exclusively in *H. pylori*. Several genes outside the *cag* PAI are also reported to be required for Cag T4SS activity (49–56). In this study, we used multiple biochemical approaches to identify proteins associated with the Cag T4SS apparatus. The results indicate that CagW, CagL, CagI, and CagH are associated with the Cag T4SS OMCC and are required for Cag T4SS activity. In contrast, none of the three known components of the Cag T4SS IMC (Cagβ, Cagα, and CagE) were identified in the current studies (35). Similarly, the non-Cag proteins previously reported to be required for Cag T4SS activity (HopQ, BabA, AlpA/B, Hyd, and HP1564) were not identified in the current studies (49–56).

CagW, CagL, CagI, and CagH were copurified with HA-CagF, but these four proteins were not copurified when targeting HA-tagged control proteins (Fig. 1; Table 1). Additionally, we detected a significantly higher relative abundance of spectral counts for CagW, CagL, CagI, and CagH in the elution from the HA-CagF immunopurification compared with the unprocessed *H. pylori* lysate (Fig. 2; Table S3). We also present evidence that CagW, CagL, CagI, and CagH co-elute with the OMCC in size exclusion chromatography experiments (Fig. 4; Table S4). Notably, we found that these four proteins were isolated from a mutant strain lacking peripheral OMCC components (HA-CagF Δ*cag3*) and a mutant strain lacking a structured outer membrane cap (HA-CagF Δ*cagM*), but they were not isolated from an OMCC assembly defective mutant (HA-CagF Δ*cagX*) or from a HA-CagF Δ*cagA* mutant (from which the OMCC was not isolated) (Table 3). Despite the association of CagW, CagL, CagI, and CagH with the Cag T4SS OMCC and the essentiality of these proteins for Cag T4SS activity, these proteins are not required for OMCC assembly (Fig. 5). The OMCCs formed by Δ*cagW*, *cagL::kan*, Δ*cagI*, or Δ*cagH* mutant strains cannot be distinguished from OMCCs formed by a wild-type strain based on the resolution of the current NS-EM analysis (Fig. 5B), but we speculate that structural differences might be detectable if high-resolution cryo-EM studies were undertaken.

Across our analyses, we consistently detected lower numbers of spectral counts corresponding to CagW, CagL, CagI, and CagH, relative to the numbers of spectral counts for the five known Cag T4SS OMCC proteins. There are multiple possible explanations for this. Within the Cag T4SS OMCC, there are 17–70 copies of each of the five known protein components (36). There may be fewer copies of CagW, CagL, CagI, and CagH within the Cag T4SS apparatus. Additionally, the Cag T4SS OMCCs that are purified by the current approach may be heterogeneous, such that not every complex contains CagW, CagL, CagI, or CagH. A third possibility is that these new proteins interact weakly or transiently with the Cag T4SS OMCC, and some of the proteins may be lost during the purification process. Finally, we note that the relative abundance of CagW, CagL, CagI, and CagH in the bacterial lysate is relatively low compared with that of the five known OMCC components.

The experiments suggested that in addition to CagW, CagL, CagI, and CagH, other Cag proteins might also be associated with the OMCC. For example, CagN was specifically isolated by the HA-CagF immunopurification and was enriched in the elution compared with the unprocessed *H. pylori* lysate (Table 1; Fig. 2). CagG was also specifically isolated in the HA-CagF immunopurification, but it was not significantly enriched in the elution (Table 1; Fig. 2). Our evaluations of CagN and CagG were limited by the detection of low numbers of spectral counts for these proteins, potentially indicating that they are produced in low levels or potentially reflecting the relatively small sizes of the proteins (corresponding to a limited number of tryptic peptides available for detection). A previous study reported that CagN interacts with CagM (a component of the OMCC) (66). Notably, CagN and CagG are not required for Cag T4SS-dependent stimulation of IL-8 production in gastric epithelial cells (43, 67).

Other Cag proteins (Cag1, Cagβ, Cagα, CagZ, CagV, CagS, CagE, and CagD) were copurified in roughly equal amounts when targeting HA-CagF or HA-tagged non-Cag control proteins in immunopurifications (Table S1). Moreover, most of these proteins were not enriched in the HA-CagF immunopurification elution compared with unprocessed *H. pylori* lysate (Table S3). Additional Cag proteins (CagU, CagQ, and CagP) were not detected in the HA-CagF immunopurified elution but were detected in the lysate, which indicates that these proteins were not copurified using the HA-CagF approach (Table S3). Finally, we did not detect two Cag proteins (Cag2, Cag4) in either the lysate or elution, which suggests that these proteins are not produced or that they are difficult to detect by mass spectrometry (Table S3).

We also identified several non-Cag proteins that were specifically associated with the HA-CagF immunopurification, enriched in the elution compared to unprocessed lysate, and/or predominantly found in high molecular mass size exclusion fractions alongside the Cag T4SS OMCC. We focused on non-Cag proteins identified by at least two of the three complementary approaches. Seven proteins were found to be significantly associated with the HA-CagF immunopurification and were significantly enriched in the elution: HP0030 (a hypothetical protein), HP0437 (TnpA, an IS605 transposase), HP0438 (TnpB, an IS605 transposase), HP0583 (a predicted flagellar biosynthesis protein), HP0747 (a predicted S-adenosylmethionine-dependent methyltransferase), HP1050 (ThrB, a homoserine kinase), and HP1279 (TrpC, an indole-3-glycerol phosphate synthase). One protein (RpsC, a ribosomal subunit component) was found to be enriched in the elution and was present predominantly in high molecular mass size exclusion fractions. Finally, one protein (HP0282, an RCK C-terminal domain-containing protein) was found to be significantly associated with the HA-CagF immunopurification, significantly enriched in the elution and predominantly present in the high molecular mass size exclusion fractions. HP0282 is predicted to bind unidentified ligands and regulate potassium, sodium, and other transporters. At present, it is not known if any of these proteins have functional roles relevant to Cag T4SS activity.

In addition to conducting immunopurifications using HA-CagF as a target, we also conducted similar immunopurifications targeting several other HA-tagged Cag proteins (Cagβ, Cagα, CagZ, CagY, CagE, and CagA). Five of these targeted proteins are localized to various subassemblies of the Cag T4SS apparatus, and CagA is a secreted effector protein. In contrast to the results obtained with immunopurification of HA-CagF, none of the immunopurifications targeting alternate tagged proteins allowed copurification of large numbers of Cag proteins (Table S2). Of particular interest, we found that copurification of OMCC components was relatively inefficient when targeting HA-CagA, despite CagA being necessary for isolation of the OMCC in the HA-CagF immunopurification (46). Notably, we were able to isolate the Cag T4SS OMCC from a different strain of *H. pylori*, strain G27, indicating that the HA-CagF immunopurification method for OMCC isolation is applicable to other *H. pylori* strains. The factors that allow for immunopurification of HA-CagF to yield the intact OMCC are still incompletely defined.

CagW, CagL, CagI, and CagH are species-specific *H. pylori* proteins that were each previously reported to be required for Cag T4SS activity (43, 65, 68, 69). Previous studies suggested that CagW is a VirB6 homolog based on a prediction of transmembrane domains (70, 71) and that CagL functions as a VirB5-like protein (72). Notably, our analyses have not detected sequence relatedness of CagW and CagL to VirB6 and VirB5 proteins, respectively, in other species. Correspondingly, the structure of CagL has been resolved using X-ray crystallography and does not share structural homology with other resolved VirB5 structures (73). Similarly, the structure of CagI has been resolved using X-ray crystallography and does not share structural homology with any known bacterial T4SS components (69). The structures of CagW and CagH have not been resolved, and AlphaFold3 predictions for these proteins have low confidence. CagW, CagL, and CagI are predicted to have amino-terminal signal sequences as determined by SignalP 6.0 analysis; correspondingly, we were unable to detect peptides corresponding to these signal sequences in our mass spectrometry analyses. CagH is not predicted to have a signal sequence based on SignalP 6.0 analysis.

CagL has been proposed to localize to pilus structures (74), and previous studies provided evidence of interactions among CagL, CagI, and CagH proteins (65, 68, 75). CagL stability has been shown to be dependent on the presence of CagI or CagH (65, 68). In the current study, we were able to isolate the OMCC from the HA-CagF Δ*cagI* mutant, but CagW, CagL, and CagH were not isolated from this strain (Table 4). This suggests that CagI may play an important role in facilitating interactions of CagW, CagL, and CagH with the OMCC. In addition, CagW, CagL, and CagH might have diminished stability in the absence of CagI (Table 4). We investigated the latter possibility by assessing the presence of CagW, CagL, and CagH in unprocessed lysate of the HA-CagF Δ*cagI* mutant and other mutant strains. The results suggested that our inability to isolate CagW, CagL, CagI, and CagH from the tested Cag mutant strains (HA-CagF Δ*cagA*, HA-CagF Δ*cagX*, HA-CagF Δ*cagI*, and HA-CagF Δ*cagH*) was not due to instability of those proteins in the mutant backgrounds (Table S5).

Our understanding of the overall structure of the Cag T4SS is still incomplete. Single-particle cryo-EM studies have identified five protein components (Cag3, CagY, CagX, CagT, and CagM) of the OMCC (34, 36, 38, 46), and cryo-ET analysis has identified at least three protein components (Cagβ, Cagα, and CagE) of the IMC (35). The composition of Cag T4SS subassemblies that potentially link the OMCC and IMC in the periplasmic region has yet to be defined. One possibility for how CagW, CagL, CagI, and CagH interface with the Cag T4SS OMCC is that they are structural components of periplasmic subassemblies such as the stalk or arches (35, 36). In the *E. coli* R388 T4SS, VirB6 and VirB5 (previously proposed to be homologs of CagW and CagL, respectively) localize to the stalk (39). We speculate that CagW, CagL, CagI, and CagH may play similar roles in the overall structure of the Cag T4SS, despite lacking sequence or structural relatedness to VirB6 or VirB5. Another site where CagW, CagL, CagI, and CagH may interact with the Cag T4SS OMCC is at the surface of the bacterium (65, 68, 69, 74). In future studies, it will be important to further define how CagW, CagL, CagI, and CagH are associated with the OMCC and further investigate how these proteins contribute to Cag T4SS activity.

## MATERIALS AND METHODS

### *H. pylori* growth conditions

*H. pylori* strains analyzed in this study are listed in Table 5. *H. pylori* strains were grown on trypticase soy agar plates containing 5% sheep blood at 37°C in room air supplemented with 5% CO_2_. Liquid cultures were grown in bisulfite-free Brucella broth supplemented with 10% heat-inactivated fetal bovine serum (FBS) in a shaking incubator under the same conditions.

**TABLE 5:** *H. pylori* strains used in this study

Strain designation	Strain name	Genotype	Reference
	HA-CagF	26695 Δ*cagF* Δ*rdxA* (Mtz^R^) *ureA::*HA-*cagF* (Chl^R^)	(46)
	VM329	26695 *hp0179*-HA (Kan^R^)	(57)
	VM333	26695 *ureA::hp0838*-HA (Chl^R^)	(57)
KB1	HA-CagF G27	G27 *ureA::*HA-*cagF* (Chl^R^)	This study
KB2	HA-Cagβ	26695 Δ*rdxA* (Mtz^R^) Δ*cagβ ureA::*HA-*cagβ* (Chl^R^)	This study
KB3	HA-Cagα	26695 Δ*rdxA* (Mtz^R^) Δ*cagα ureA::*HA-*cagα* (Chl^R^)	This study
KB4	CagZ-HA	26695 *ureA::cagZ*-HA (Chl^R^)	This study
KB5	CagY-HA	26695 *cagY*-HA(1835) (Chl^R^)	This study
KB6	HA-CagE	26695 Δ*rdxA* (Mtz^R^) Δ*cagE ureA::*HA-*cagE* (Chl^R^)	This study
KB7	HA-CagA	26695 Δ*rdxA* (Mtz^R^) HA-*cagA*	This study
	ΔPAI	26695 Δ(*cagA-cag1*) (Chl^R^)	(76)
	HA-CagF Δ*cagX*	26695 Δ*rdxA* (Mtz^R^) Δ*cagX ureA::*HA-*cagF* (Chl^R^)	(46)
KB8	HA-CagF Δ*cagW*	26695 Δ*rdxA* (Mtz^R^) Δ*cagW ureA::*HA-*cagF* (Chl^R^)	This study
KB9	HA-CagF *cagL::kan*	26695 *cagL::kan* (Kan^R^) *ureA::*HA-*cagF* (Chl^R^)	This study
KB10	HA-CagF Δ*cagI*	26695 Δ*rdxA* (Mtz^R^) Δ*cagI ureA::*HA-*cagF* (Chl^R^)	This study
KB11	HA-CagF Δ*cagH*	26695 Δ*rdxA* (Mtz^R^) Δ*cagH ureA::*HA-*cagF* (Chl^R^)	This study
	HA-CagF Δ*cag3*	26695 Δ*rdxA* (Mtz^R^) Δ*cag3 ureA::*HA-*cagF* (Chl^R^)	(46)
	HA-CagF *cagA::kan*	26695 *cagA::kan* (Kan^R^) *ureA::*HA-*cagF*	(46)

### *H. pylori* strain generation

The construction of a marked *cagL::kan* strain and unmarked Δ*cagI* and Δ*cagH* strains was previously reported (65). The unmarked Δ*cagW* strain was generated for the current study, using counterselection methods with a *cat-rdxA* cassette (65). To generate *H. pylori* strains to produce HA-CagF, we used a modified pAD-1 plasmid to introduce a full-length copy of the *cagF* gene along with a sequence encoding an HA-epitope tag at the CagF N-terminus into the *ureAB* chromosomal locus as previously described, selecting for chloramphenicol-resistant transformants. This approach was used for genetic manipulation of the wild-type strain G27, an unmarked Δ*cagW* strain, a marked Δ*cagL* strain, an unmarked Δ*cagI* strain, and an unmarked Δ*cagH* strain.

The construction of the unmarked Δ*cagβ*, Δ*cagα*, and Δ*cagE* strains was previously reported (47). To generate *H. pylori* strains producing HA-tagged forms of other Cag proteins, we used a modified pAD-1 plasmid to introduce a full-length copy of *cagβ*, *cagα*, *cagZ*, or *cagE* along with a sequence encoding an HA-epitope tag at the N-terminus (*cagβ*, *cagα,* and *cagE*) or C-terminus (*cagZ*) into the *ureAB* chromosomal locus, selecting for chloramphenicol-resistant transformants. This approach was used for genetic manipulation of an unmarked Δ*cagβ* strain, an unmarked Δ*cagα* strain, the wild-type strain 26695, or an unmarked Δ*cagE* strain.

To generate an *H. pylori* strain producing CagY-HA from the *cagY* locus, we used a modified pUC57 plasmid to introduce a sequence encoding an HA-tag after CagY amino acid 1835 and a chloramphicol resistance gene downstream of *cagY* into wild-type strain 26695, selecting for chloramphenicol-resistant transformants. To generate an *H. pylori* strain producing HA-CagA from the *cagA* locus, we first inserted a *cat-rdxA* cassette near the *cagA* start site into a metronidazole-resistant (Δ*rdxA*) strain of 26695, selecting for chloramphenicol-resistant and metronidazole-sensitive transformants. We then used a modified pUC57 plasmid to introduce a sequence encoding an HA-tag at the CagA N-terminus, selecting metronidazole-resistant transformants.

### Immunopurification of *H. pylori* proteins

The immunopurification protocol used in this study was adapted from the previously described methods (34, 36, 46). *H. pylori* strains were grown in 125 mL liquid culture for 20 h (resulting in optical density values at 600 nm [OD_600_] between 0.9 and 1.2). Bacterial cells were pelleted at 5,000 × *g* for 15 min. The cells were resuspended in radioimmunoprecipitation assay (RIPA) buffer (0.2 M HEPES, 0.3 M NaCl, 1% NP-40, 0.25% sodium deoxycholate, pH 7.0) supplemented with 1 mM phenylmethylsulfonyl fluoride (PMSF) and protease inhibitor cocktail [cOmplete Mini, Roche]. For experiments described in Tables 3 and 4, the bacterial lysis protocol was modified so that cells were first resuspended in 0.2 M HEPES pH 7.0, 5000 U mutanolysin (Sigma-Aldrich), protease inhibitor cocktail tablet, and 5 U DNaseI (NEB), and 2× RIPA buffer with PMSF was added after 30 min of incubation. Bacterial suspensions were sonicated on ice [25% amplitude, 10 s on/10 s off × 4] and incubated for 1 h at 4°C. Bacterial lysates were centrifuged [12,000 × *g* for 15 min at 4°C] to pellet insoluble material. Monoclonal anti-HA antibodies were noncovalently linked to protein G Dynabeads (Invitrogen) and incubated with clarified bacterial lysate for 30 min at room temperature. Beads were washed three times using RIPA buffer containing 0.025% sodium deoxycholate, and proteins were selectively eluted with RIPA wash buffer containing 200 µg/mL HA peptide (YPYDVPDYA; GenScript).

### Mass spectrometry analysis

To analyze the protein content of the immunopurified samples, tryptic peptides were generated using S-Trap (Protifi), according to the manufacturer’s recommended protocol. Peptides were resuspended in 40 µL 0.2% formic acid with 0.015% n-Dodecyl-β-D-maltoside (DDM). Data-Dependent Acquisition with Parallel Accumulation-Serial Fragmentation (DDA-PASEF) data was acquired on 1 µL of peptides using a 30-min aqueous to organic gradient method delivered via a nanoELUTE2 on a PepSep 75 µm internal diameter, 25 cm length, and 1.5 µm particle size coupled to a timsTOF HT (Bruker) using a 20 µm Captive Spray emitter. DDA-PASEF data were collected in 5 PASEF ramps from 0.7 to 1.3 1 /k0 covering m/z windows from 300 to 1,700 with 100 accumulation times in the TIMS cell. Peptide MS/MS spectra were queried against an *H. pylori* protein database (strain 26695) (77) using FragPipe 19 with default parameters (78), filtered using the included implementation of Percolator to 0.1% false discovery rate (FDR), and then visualized using Scaffold 5 (Proteome Software).

Spectral count data for immunopurifications of HA-CagF 26695 (*n* = 5) and spectral count data from control immunopurifications (HP0179-HA, *n* = 2; HP0838, *n* = 3) were normalized based on the mean of the total spectral counts. Average interaction probabilities of prey proteins with HA-CagF were determined using SAINTexpress analysis (58, 59).

Spectral count data for paired lysate and elution samples from immunopurification of HA-CagF 26695 (*n* = 3) were normalized within pairs based on the mean of the total spectral counts. The protein content of lysate samples was quantified by a Pierce BCA Protein assay (ThermoScientific) following the manufacturer’s protocol. Statistical significance was determined using a paired *t*-test, with an alternative hypothesis that the true mean difference is less than zero.

To analyze the protein content of the lysate samples as described in Table S5, diaPASEF data were acquired on 50 ng of peptides using a 30 min aqueous to organic gradient method delivered via a nanoELUTE2 on a PepSep column with 75 µm internal diameter, 25 cm length, and 1.5 µm particle size coupled to a timsTOF Ultra instrument (Bruker) using a 20 µm Captive Spray emitter. diaPASEF data were collected in 12 PASEF ramps from 0.75 to 1.3 1 /k0 covering 350 to 1,250 m/z via variable windows ranging in size from 12.27 to 122.81 Th with 50 ms accumulation time. Data were searched using a Spectronaut library free search against the *H. pylori* 26695 database (77).

### CagA translocation assay

The CagA translocation assay was performed as described previously (76). AGS gastric epithelial cells were cultured in RPMI media (Corning) supplemented with 10% heat-inactivated FBS and 0.1% antibiotic-antimycotic (ThermoScientific). AGS cells were seeded in a 6-well tissue culture-treated plate at 6.0 × 10^5^ cells/well and cultured overnight at 37°C in room air supplemented with 5% CO_2_. The following day, *H. pylori* in fresh RPMI medium was added at a multiplicity of infection (MOI) of 100:1, and co-cultures were incubated for 4 h at 37°C. Cells were lysed in lysis buffer (50 mM Tris-HCl pH 7.4, 150 mM NaCl, 1% NP-40, 2 mM NaOV_4_, protease inhibitor cocktail [cOmplete Mini, Roche], and phosphatase inhibitor cocktail [PhosSTOP, Roche]). Protein content was quantified by a Pierce BCA Protein assay (ThermoScientific) following the manufacturer’s protocol. Samples (30 µg of protein) were loaded onto a 7.5% acrylamide gel (Mini-PROTEAN TGX, Bio-Rad), separated by SDS-PAGE, and transferred to a 0.22 µm nitrocellulose membrane. The membranes were immunoblotted with anti-phosphotyrosine monoclonal antibody (PY99, Santa Cruz Biotechnology) or previously described anti-CagA polyclonal antiserum (79). Immunoreactive bands were visualized using either a peroxidase-conjugated goat anti-mouse IgG or goat-anti-rabbit IgG (Promega) and enhanced chemiluminescence.

### Size exclusion chromatography

The elution sample from an HA-CagF immunopurification (prepared as described above) was diluted to 0.5 mL in RIPA buffer containing 0.025% sodium deoxycholate and fractionated using a Superose 6 10 × 300 mm column, equilibrated in the same buffer, at a flow rate of 0.5 mL/min; 1 mL fractions were collected from 0.2 to 1.25 column volume (CV) (25 mL) and stored at −20°C.

### Western blot analysis

Immunopurified proteins were separated by SDS-PAGE using a 4%–20% gradient gel (Mini-PROTEAN TGX, Bio-Rad). The proteins were transferred to a 0.22 µm nitrocellulose membrane. The membranes were immunoblotted with previously described rabbit polyclonal antisera to *H. pylori* Cag proteins (76). Immunoreactive bands were visualized using peroxidase-conjugated goat anti-rabbit IgG (Promega) and enhanced chemiluminescence.

### IL-8 induction assay

For IL-8 assays, AGS cells were seeded in a 96-well tissue culture-treated plate at 2.5 × 10^4^ cells/well and cultured overnight at 37°C in room air supplemented with 5% CO_2_. The following day, *H. pylori* in fresh RPMI medium was added at an MOI of 100:1, and co-cultures were incubated for four hours at 37°C. The co-culture supernatants were collected and stored at −20°C overnight. IL-8 content was quantified using a human CXCL8 enzyme-linked immunosorbent assay (R&D Systems) following the manufacturer’s protocol (46).

### Negative stain electron microscopy analysis

Samples for negative stain electron microscopy were prepared as previously described (80). In total, 3.5 µL aliquots were absorbed twice into a glow-discharged 400-mesh copper grid covered with carbon-coated collodion film (Electron Microscopy Sciences). Grids were washed in two drops of water and stained with two drops of uranyl formate (0.75%). Samples were imaged on a 100 kV Morgagni electron microscope (ThermoFisher) at a magnification of 22,000× with an Orius SC200 CCD camera (2.5 Å/pixel).

## SUPPLEMENTAL MATERIAL

Fig. S1 iai.00493-24-s0001.pdf Translocation of HA-CagA.

Supplemental tables iai.00493-24-s0002.xlsx Tables S1 to S5.

ASM does not own the copyrights to Supplemental Material that may be linked to, or accessed through, an article. The authors have granted ASM a non-exclusive, world-wide license to publish the Supplemental Material files. Please contact the corresponding author directly for reuse.
