# Supplementary material for: Species-specific components of the Helicobacter pylori Cag type IV secretion system
Source: Infect Immun. 2025 Apr 10;93(5):e00493-24. doi: 10.1128/iai.00493-24 (PMC12070742; doi:10.1128/iai.00493-24)
Supplement: Fig. S1 — Translocation of HA-CagA. [file iai.00493-24-s0001.pdf]

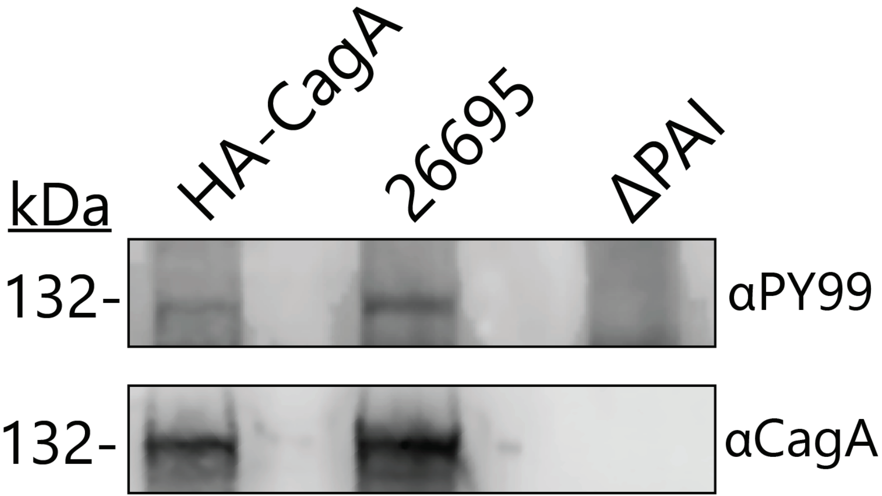

Supplementary Figure 1. Translocation of HA-CagA. The indicated strains were co-cultured with AGS cells and a CagA translocation assay was performed as described in the Methods section. Protein samples were analyzed by Western blotting using anti-phosphotyrosine (PY99) antibody or anti-CagA antiserum.
